# Updated cost-effectiveness analysis of tislelizumab in combination with chemotherapy for the first-line treatment of advanced gastric cancer or gastroesophageal junction adenocarcinoma

**DOI:** 10.3389/fonc.2024.1477722

**Published:** 2024-12-16

**Authors:** Lei Xu, Yunchun Long, Lu Yao, Hao Wang, Weihong Ge

**Affiliations:** ^1^ Department of Pharmacy, Nanjing Drum Tower Hospital Clinical College of Nanjing University of Chinese Medicine, Nanjing, Jiangsu, China; ^2^ Department of Pharmacy, Meishan People ‘s Hospital, Meishan, Sichuan, China; ^3^ Department of Pharmacy, Nanjing Drum Tower Hospital Clinical College of Xuzhou Medical University, Xuzhou, Jiangsu, China

**Keywords:** tislelizumab, advanced gastric cancer, gastroesophageal junction adenocarcinoma, cost-effectiveness, partition survival model, chemotherapy

## Abstract

**Objective:**

The RATIONALE-305 trial demonstrated that tislelizumab in combination with chemotherapy regimens was more beneficial than chemotherapy regimens alone in the treatment of patients with advanced gastric cancer or gastroesophageal junction adenocarcinoma (GC/GEJC). This study aimed to evaluate the cost-effectiveness of tislelizumab combination chemotherapy in the treatment of advanced GC/GEJC from the perspective of the Chinese health service system.

**Methods:**

A three-state partition survival model was constructed to evaluate the economics of tislelizumab combined with chemotherapy as the first-line treatment of advanced GC/GEJC. Clinical data were collected from the RATIONALE-305 trial, and the incremental cost-effectiveness ratio (ICER) was calculated using quality-adjusted life years (QALYs) as the output index. The stability of the results was verified using sensitivity and subgroup analyses. In addition, scenario analysis was conducted for the model simulation time and different parameter extrapolation models.

**Results:**

The results of basic analysis showed an increase of 0.31 QALYs in the tislelizumab group compared with the placebo group (1.53 QALYs *vs* 1.22 QALYs), and a concomitant increase in cost of 10,326.68 USD, with an ICER of 33,876.38 USD/QALY, which is less than the current Chinese willingness-to-pay threshold (36,924.80 USD/QALY). Sensitivity analyses demonstrated that the utility values of progression-free survival, progressive disease and the price of capecitabine had a greater impact on the model. Subgroup analysis revealed that combination therapy was equally cost-effective in people with a program death ligand 1 tumor area positivity score of ≥5%.

**Conclusion:**

From the perspective of the Chinese health service system, the treatment of advanced GC/GEJC with tislelizumab combined with chemotherapy has a cost-effective advantage over chemotherapy alone.

## 1 Introduction

According to the GLOBOCAN 2022 data, there are more than 970,000 new cases and approximately 660,000 deaths from gastric cancer (GC) worldwide, and its morbidity and mortality rank fifth among all cancers (1). GC is one of the main malignant tumors in China. The latest statistical data show that it ranks fifth and third in morbidity and mortality, respectively (2). Although the overall incidence of GC is declining, it is gradually increasing among young people, and the number of new GC cases is expected to increase in the future (3).

GC is already in the progressive or advanced stages when most patients are diagnosed, owing to the lack of clear clinical indications, and the prognosis of patients with advanced GC is poor, with a 5-year survival rate of approximately 10% (4). Fluorouracil combined with platinum-based chemotherapy is the first-line therapeutic option for the treatment of advanced GC or gastroesophageal junction adenocarcinoma (GEJC); however, its efficacy is less than satisfactory, and the median overall survival (mOS) of patients is shorter, not more than one year in most cases (5–7). Therefore, it is particularly important to identify treatments that can significantly prolong the survival of patients with advanced GC/GEJC and improve their prognosis.

Current breakthroughs in immunotherapy have brought hope to patients with advanced GC/GEJC (8). Both the CheckMate-649 and KEYNOTE-859 trials validated that programmed death 1 (PD-1) inhibitors in combination with chemotherapy significantly prolonged the median progression-free survival and mOS (9). Tislelizumab is a humanized Immunoglobulin G4 (IgG4) anti-PD-1 monoclonal antibody. RATIONALE-305 is a global phase III clinical trial that aimed to study the efficacy and safety of tislelizumab plus chemotherapy as a first-line treatment in patients with advanced GC/GEJC (10). The results of the study showed that tislelizumab combination chemotherapy significantly improved mOS (15 months *vs* 12.9 months), reduced the risk of death by 20%, achieved a 2-year survival rate of 33%, and the safety was controllable, with the regimen resulting in an incidence of grade 3 and higher adverse reactions of 53.8%. This was lower than that of other similar phase III clinical studies of immune combined chemotherapy for advanced GC/GEJC.

The choice of a drug should be based not only on its safety and efficacy but also on its economy. Tislelizumab, in combination with chemotherapy, has a significant survival benefit; however, its economic benefits remain unknown. Therefore, this study evaluated the cost-effectiveness of tislelizumab combined with chemotherapy for advanced GC/GEJC treatment from the perspective of China’s health service system.

## 2 Materials and methods

### 2.1 Target population and treatment programs

The target population for this study was consistent with that of the RATIONALE-305 clinical trial, which included patients aged 18 years or older with histologically confirmed locally advanced unresectable or metastatic untreated adenocarcinoma of the GC/GEJC without systemic treatment, with an Eastern Cooperative Oncology Group physical status score of 0 or 1.

All eligible patients received up to six cycles of chemotherapy, with one cycle every three weeks. The chemotherapy regimen was as follows: capecitabine (1000 mg/m^2^ twice daily on days 1–14 of each cycle) plus oxaliplatin (130 mg/m^2^ on day 1 of each cycle) or 5-fluorouracil (800 mg/m^2^ on days 1–5 of each cycle) plus cisplatin (80 mg/m^2^ on day 1 of each cycle). The tislelizumab group received tislelizumab every three weeks in addition to chemotherapy (200 mg on day 1 of each cycle). According to the RATIONALE-305 trial, 93% of patients in the tislelizumab group were treated with capecitabine plus oxaliplatin and 7% with 5-fluorouracil plus cisplatin, whereas 93.75% of patients in the placebo group were treated with capecitabine plus oxaliplatin and 6.25% with 5-fluorouracil plus cisplatin. Patients initially treated with capecitabine plus oxaliplatin chemotherapy received capecitabine maintenance therapy until disease progression. The RATIONALE-305 trial revealed that after disease progression, 53% of patients in the tislelizumab group and 59% of patients in the placebo group received follow-up anticancer therapy, whereas those who did not receive follow-up received optimal supportive care. However, because follow-up treatment was not provided in the trial as a specific regimen, irinotecan (125 mg/m^2^ on days 1 and 8 of each cycle) was selected as the drug after disease progression in this study according to the recommendations of the Chinese Society of Clinical Oncology Guidelines for the Diagnosis and Treatment of Gastric Cancer 2023 (5).

### 2.2 Model structure

**Figure 1:**
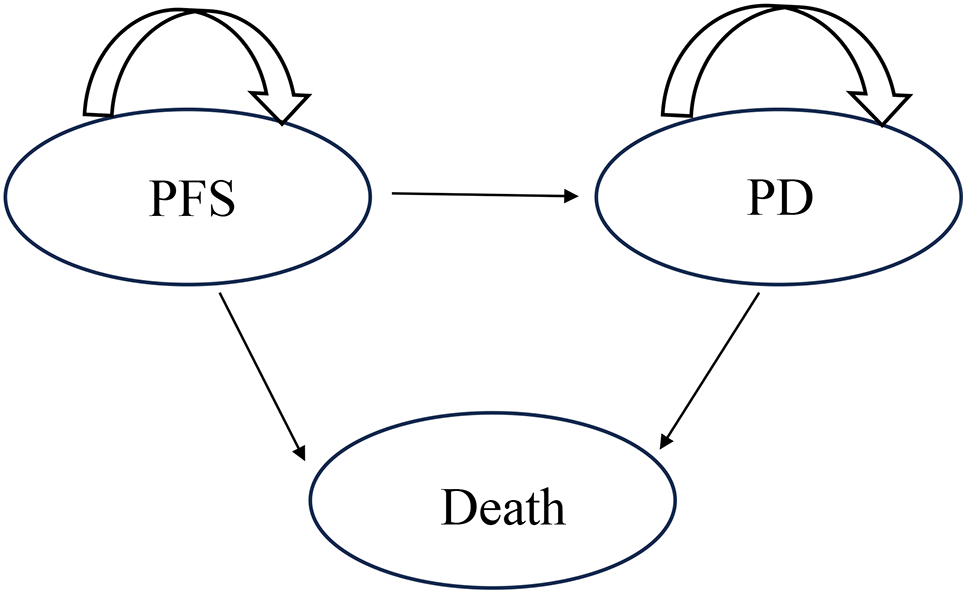
**A** three-state partitioned survival model to simulate advanced gastric cancer or gastroesophageal junction adenocarcinoma. PFS, progression-free survival; PD, progressive disease.

The TreeAge Pro software (2022 edition) was used to construct a partition survival model. The model was divided into three states: progression-free survival (PFS), progressive disease (PD), and death (**Figure 1**). It was assumed that all patients were in the PFS state when they entered the model, with 3 weeks as a cycle. Given the low 5-year survival rate and poor prognosis of patients with advanced GC/GEJC, published studies on advanced GC/GEJC typically used 10 years as the model simulation time (11–13). Therefore, the simulation timeframe for this study was set as 10 years. The main results of the model outputs were the total cost, quality-adjusted life-years (QALYs), and cost-effectiveness ratio (ICER). According to the recommendations of the World Health Organization and the Guidelines for the Evaluation of Pharmacoeconomics in China (2020), three times the per capita gross domestic product of China in the year 2023 was used as the willingness-to-pay (WTP) threshold (WTP=36,924.80 USD/QALY); additionally, the cost and utility values were discounted at an annual discount rate of 5% (14).

### 2.3 Survival analysis

**Figure 2:**
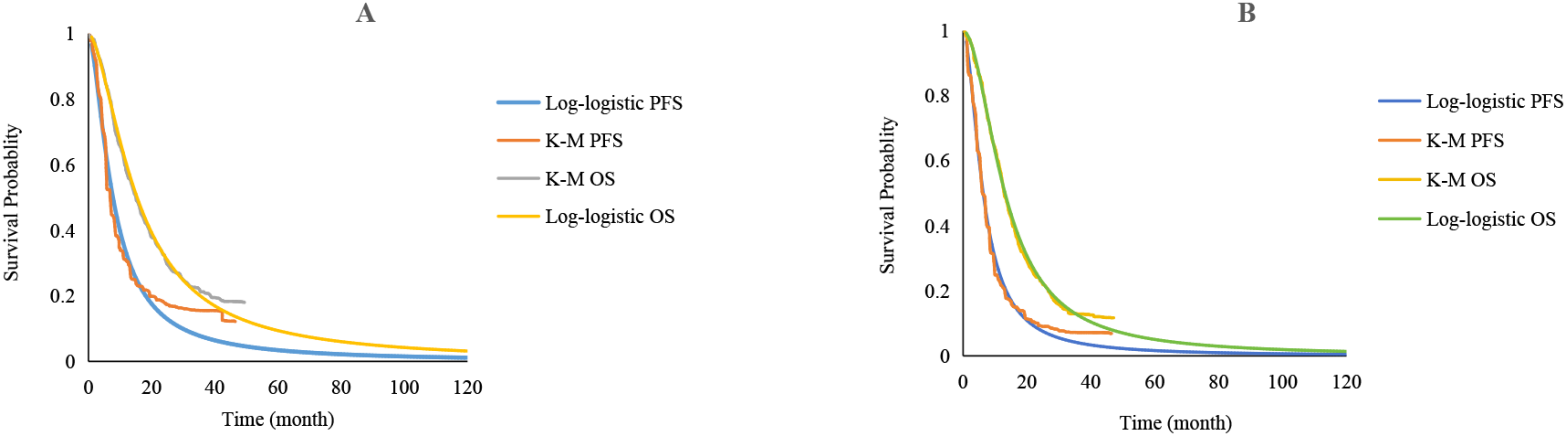
Extrapolation of Kaplan-Meier (K-M) curves for standard parametric models. **(A)** PFS and OS curves of the tislelizumab group; **(B)** PFS and OS curves of the placebo group; K-M, Kaplan-Meier; PFS, progression-free survival; OS, overall survival.

In this study, the Engauge Digitizer software (version 11.1) was used to extract the data points on the Kaplan-Meier (K-M) curve in the RATIONALE-305 trial, and the survHE package in R language 4.4.0 software was utilized to reconstruct the individual patient data according to the survival rate, time, sample size, and number of risks for high-risk groups (15). Exponential, Weibull, Gompertz, Log-logistic, Log-normal, and Gamma distributions were used to fit the survival curves. The best-fitting distribution was then selected according to the Akaike information criterion and Bayesian information criterion, and combined with visual inspection. A log-logistic distribution was used to fit PFS and overall survival (OS) curves. The fitted parameters and curves are presented in **Table 1** and **Figure 2**, respectively.

**Table 1:** Fitting parameters for the Kaplan-Meier (K-M) curve.

	PFS of tislelizumab group		PFS of placebo group		OS of tislelizumab group		OS of placebo group	
	AIC	BIC	AIC	BIC	AIC	BIC	AIC	BIC
Exponential	2593.38	2597.60	2592.24	2596.45	3035.45	3039.66	3140.49	3144.70
Weibull	2595.35	2603.78	2588.69	2597.11	3021.25	3029.69	3100.02	3108.44
Gompertz	2556.06	2564.49	2583.24	2591.65	3036.91	3045.34	3133.45	3141.86
Log-logistic	2491.35	2499.78	2497.42	2505.84	2989.46	2997.90	3066.77	3075.18
Log-normal	2500.04	2508.47	2499.78	2508.19	2995.81	3004.25	3081.03	3089.45
Gamma	2591.75	2600.19	2575.15	2583.56	3013.31	3021.74	3087.53	3095.95

PFS, progression-free survival; OS, overall survival; Tislelizumab group, tislelizumab plus chemotherapy; Placebo group, placebo plus chemotherapy; AIC, Akaike information criterion; BIC, Bayesian information criterion.

### 2.4 Cost and utility value

Our study only considered direct medical costs, including drug use, follow-up, hospitalization, and optimal supportive care costs. Drug costs were obtained from the latest average prices published on the China Medical Information Network (https://www.menet.com.cn); follow-up costs, hospitalization costs, and optimal supportive care costs were obtained from the published literature (16, 17); The cost of treatment for adverse drug reactions (ADRs) was calculated by multiplying the incidence of ADRs by the cost of treatment for a single ADR, and the incidence of ADR was obtained from the RATIONALE-305 study (10). To simplify the model, we considered only grade 3–5 serious adverse reactions and calculated them only in the first cycle. Some of the drugs in the treatment regimen must be administered based on body weight and Body Surface Area (BSA); therefore, in this study, it was assumed that the patient’s body weight was 65 kg and BSA was 1.72 m^2^ (18).

Since the RATIONALE-305 study did not specify health-related quality of life, the health status utility values used in this study were derived from the published literature. The utility values of PFS and PD were 0.797 and 0.577, respectively (19). Additionally, the negative utility values associated with adverse reactions also came from the published literature (20, 21). The specific parameters are listed in **Table 2**.

**Table 2:** Model cost and utility parameters.

Parameters	Baseline values	Low values	High values	Distribution	Source
Cost (USD)					
Tislelizumab per 100mg	181.20	172.66	189.74	Gamma	Menet
Capecitabine per 500mg	0.34	0.10	3.03	Gamma	Menet
Oxaliplatin per 50mg	38.51	5.07	242.42	Gamma	Menet
5-fluorouracil per 250mg	6.09	1.32	16.39	Gamma	Menet
Cisplatin per 30mg	5.20	1.28	10.47	Gamma	Menet
Irinotecan per 100mg	129.59	111.84	131.81	Gamma	Menet
Anemia per cycle	5.27	4.21	6.32	Gamma	Local price
Platelet count decreased per cycle	1156.34	925.07	1387.61	Gamma	Local price
Neutrophil count decreased per cycle	15.84	12.67	19.01	Gamma	Local price
Neutropenia per cycle	63.64	76.36	50.91	Gamma	Local price
Hospitalization per cycle	61.31	49.04	73.57	Gamma	(16)
Follow-up per cycle	80.71	64.57	96.85	Gamma	(17)
CT per cycle	141.29	113.03	169.55	Gamma	(17)
Optimal supportive care per cycle	164.57	131.66	197.48	Gamma	(17)
Incidence of adverse reactions					
Tislelizumab plus chemotherapy					
Anemia	0.050	0.040	0.060	Beta	(10)
Platelet count decreased	0.110	0.088	0.132	Beta	(10)
Neutrophil count decreased	0.120	0.096	0.144	Beta	(10)
Neutropenia	0.070	0.056	0.084	Beta	(10)
Placebo plus chemotherapy					
Anemia	0.070	0.056	0.084	Beta	(10)
Platelet count decreased	0.120	0.096	0.144	Beta	(10)
Neutrophil count decreased	0.120	0.096	0.144	Beta	(10)
Neutropenia	0.070	0.056	0.084	Beta	(10)
Utility value					
PFS status	0.797	0.638	0.956	Beta	(19)
PD status	0.577	0.462	0.692	Beta	(19)
Negative utility value					
Anemia	0.073	0.058	0.088	Beta	(20)
Platelet count decreased	0.023	0.018	0.028	Beta	(21)
Neutrophil count decreased	0.200	0.160	0.240	Beta	(20)
Neutropenia	0.200	0.160	0.240	Beta	(20)
Others					
Weight/kg	65	52	**7**2	Normal	(18)
BSA/m^2^	1.720	1.376	2.060	Normal	(18)
Discount rate/%	5	0	8	Beta	(14)

PFS, progression-free survival; PD, progressive disease; BSA, Body surface area.

### 2.5 Sensitivity analysis

In this study, a one-way sensitivity analysis (OWSA) and probabilistic sensitivity analysis (PSA) were used to assess the robustness of the model. The OWSA was performed to determine the drug cost, utility value, and incidence of adverse reactions, and the results are presented in a tornado diagram. The upper and lower bounds of drug cost were obtained from Menet, the ranges of PFS and PD status utility values were obtained from the literature (17), and the upper and lower bounds of all other parameters fluctuated within ±20% of their base values. PSA was performed using a second-order Monte Carlo simulation with 1000 random samplings. All cost data were gamma distributed and utility values and incidence of adverse effects were beta distributed to analyze the effect of simultaneous changes in multiple parameters on the results, and finally, the results of the study were presented through cost-effect scatter plots and cost-effect acceptable curves.

### 2.6 Subgroup analysis and scenario analysis

The RATIONALE-305 trial provided K-M curve for patients with a program death ligand 1 (PD-L1) tumor area positivity (TAP) score of ≥5%, and considering that different cost-effectiveness outcomes may occur in subgroup populations, for this reason, we used a base analysis to analyze cost-effectiveness in patients with a PDL-1 TAP score of ≥5%.

In the scenario analysis, we simulated patient survival times of 5 and 20 years to explore the survival benefits for patients over the entire course of the disease. In addition, the OS curves for tislelizumab plus chemotherapy and placebo plus chemotherapy were fitted and extrapolated using the Royston/Parmar spline, non-mixture cure, and mixture cure models to assess whether fitting extrapolated survival curves with different models would have an impact on the final outcome.

## 3 Results

### 3.1 Basic analysis results

The results of the basic analysis are shown in **Table 3**, which shows an increase in 0.31 QALYs in the tislelizumab group compared with the placebo group, with a consequent increase in total cost of 10,326.68 USD, and a lower ICER (33,876.38 USD/QALY) than the WTP (36,924.80 USD/QALY). Therefore, the tislelizumab combination chemotherapy regimen has a relative advantage.

**Table 3:** Results of the basic analysis.

Parameters	Total cost (USD)	Incremental cost (USD)	QALYs	Incremental QALYs	ICER (USD/QALY)
Placebo group	16266.59		1.22		
Tislelizumab group	26593.27	10326.68	1.53	0.31	33876.28

Placebo group, Placebo plus chemotherapy; Tislelizumab group, Tislelizumab plus chemotherapy; QALYs, Quality adjusted life years; ICER, Incremental cost-effectiveness ratio.

### 3.2 Sensitivity analysis results

**Figure 3:**
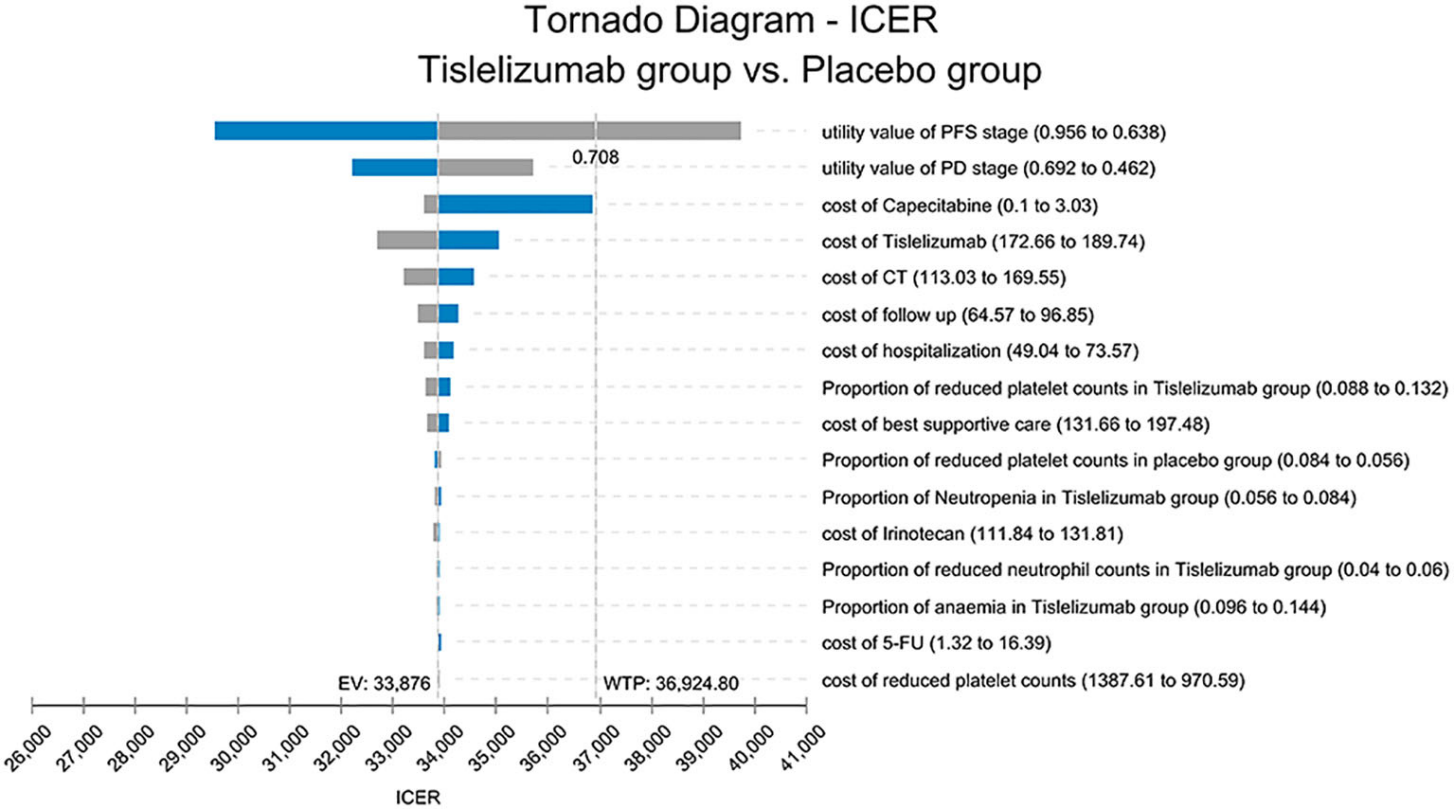
The tornado chart of One-way sensitivity analysis. ICER, Incremental cost-effectiveness ratio; PFS, progression-free survival; PD, progressive disease; Tislelizumab group, tislelizumab plus chemotherapy; Placebo group, placebo plus chemotherapy; EV, expected value; WTP, willingness-to-pay.

The OWSA results are shown in **Figure 3**, where PFS, PD state utility values, and capecitabine price had the greatest impact on ICER; the lower the PFS utility value, the higher the ICER. When the value is close to its lower limit, the ICER is greater than the WTP, leading to the opposite result. Factors such as tislelizumab, CT, and the cost of follow-up also had an impact on the model but did not influence the final results.

**Figure 4:**
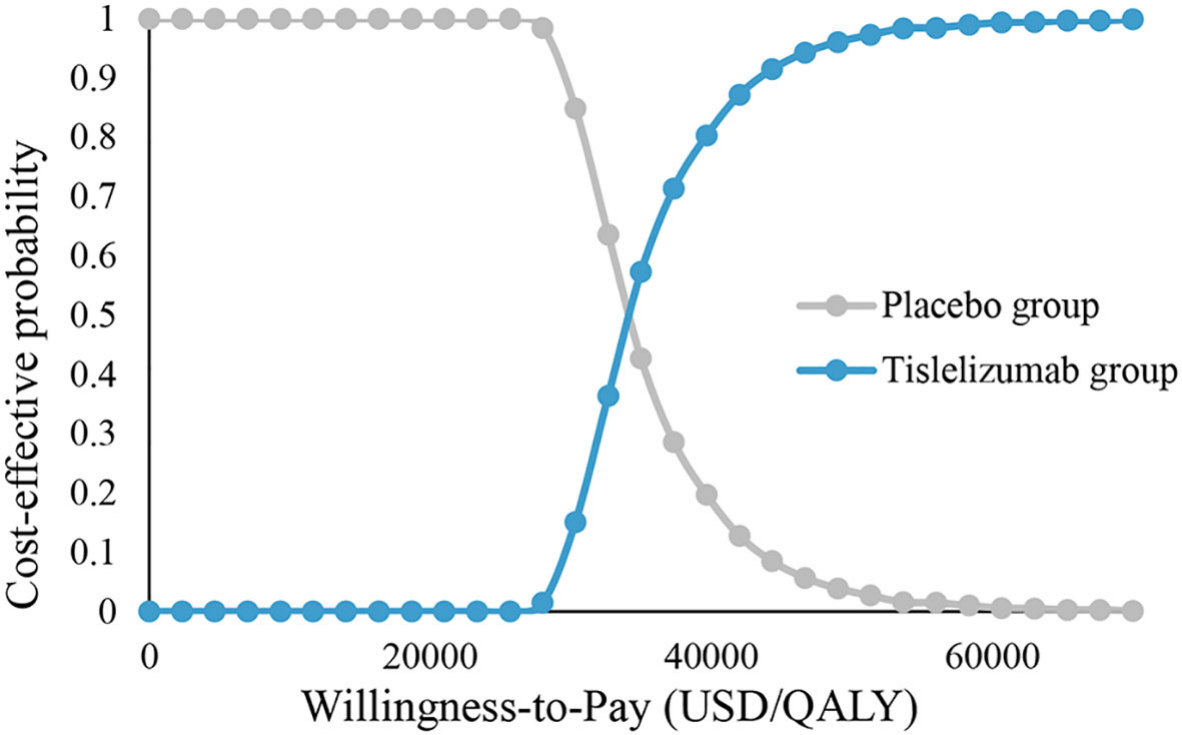
Cost-effectiveness acceptability curves. Tislelizumab group, tislelizumab plus chemotherapy; Placebo group, placebo plus chemotherapy; QALY, Quality adjusted life year.

**Figure 5:**
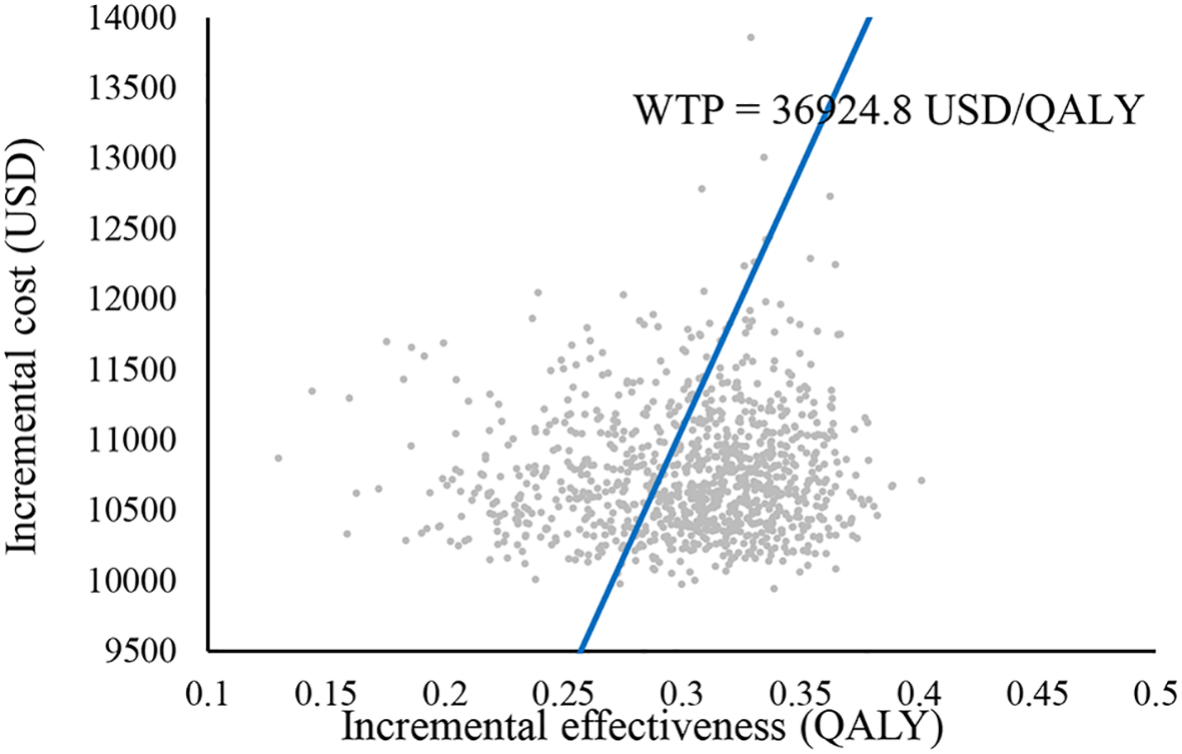
Cost-effectiveness scatter plot. WTP, willingness-to-pay; QALY, Quality adjusted life year.

The PSA results are shown in **Figures 4**, **5**. **Figure 4** shows that tislelizumab combination chemotherapy started to be cost-effective when the WTP was 28,000 USD/QALY, the probability of tislelizumab combination chemotherapy being cost-effective was 50% when the WTP was 34,333.3 USD/QALY, and the probability of being cost-effective gradually increased with the increase of WTP, when the WTP was 51,333.3 USD/QALY, the probability of being cost-effective increased to 97%. **Figure 5** shows that 673 points lie below the WTP line; therefore, the probability that tislelizumab in combination with chemotherapy is an economically superior regimen is 67.3%.

### 3.3 Subgroup analysis and scenario analysis results

The results of the subgroup analysis are shown in **Table 4**, where receiving the tislelizumab combination chemotherapy regimen in patients with a PD-L1 TAP score of ≥5% was associated with a cost increase of 11,542.55 USD over receiving the placebo combination chemotherapy regimen, yielding an additional 0.43 QALYs with an ICER of 26,506.06 USD/QALY. compared to the ‘3.1’ under the full sample analysis pool of GC/GEJC patients, this regimen was more economical when applied to the subgroup of patients with a PDL-1 TAP score of ≥5%.

**Table 4:** Results of subgroup and scenario analyses.

Parameters	Total cost (USD)	Incremental cost (USD)	QALYs	Incremental QALYs	ICER (USD/QALY)	
Subgroup analysis						
Placebo group	16856.86		1.19			
Tislelizumab group	28399.42	11542.55	1.62	0.43	26506.06	
Scenario analysis						
5 years						
Placebo group	15014.81		1.14			
Tislelizumab group	23534.95	8520.14	1.35	0.21	39811.96	
20 years						
Placebo group	16928.06		1.27			
Tislelizumab group	28601.47	11673.41	1.64	0.37	31190.51	
Royston/Parmar spline model						
Placebo group	16270.55		1.22			
Tislelizumab group	26598.41	10327.87	1.53	0.30	33872.73	
Mixture cure model						
Placebo group	21296.67		1.47			
Tislelizumab group	30588.89	9292.21	1.73	0.26	35560.10	
Non-mixture cure model						
Placebo group	21383.81		1.47			
Tislelizumab group	31264.40	9880.59	1.76	0.29	33926.13	

Placebo group, Placebo plus chemotherapy; Tislelizumab group, Tislelizumab plus chemotherapy; QALYs, Quality adjusted life years; ICER, Incremental cost-effectiveness ratio.

The results of the scenario analysis showed that when the model simulated survival time was adjusted to 5 and 20 years, the ICER of the tislelizumab group was 39,811.96 USD/QALY and 31,190.51 USD/QALY, respectively, which shows that there was a decreasing trend in the ICER with the prolongation of the simulated survival time.

**Figure 6:**
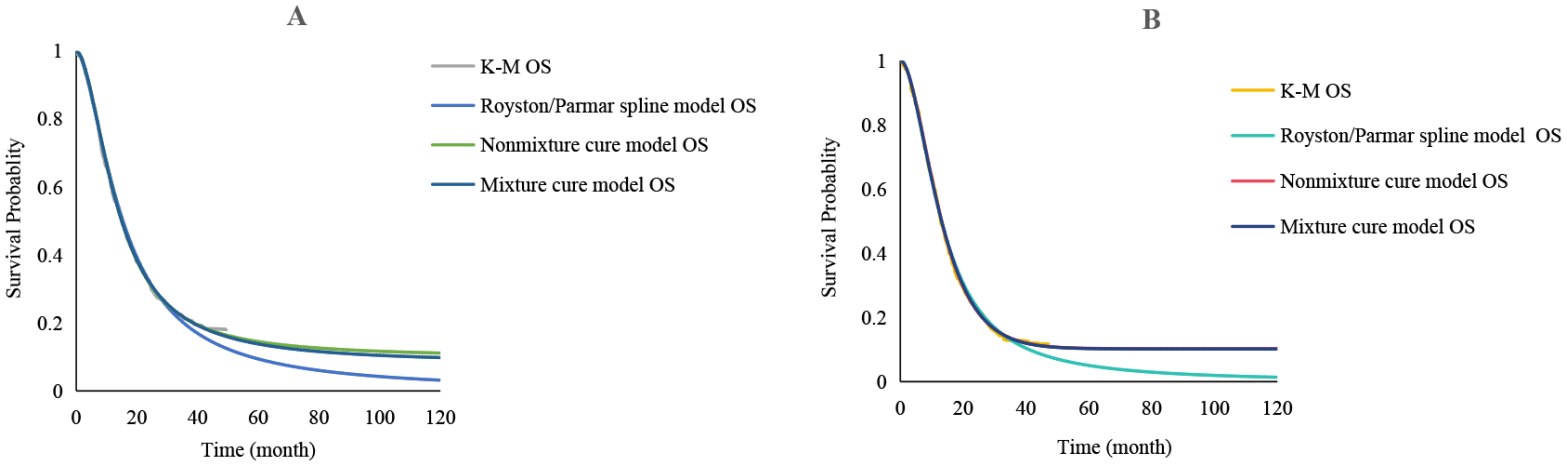
Royston/Parmar spline, mixture cure, and non-mixture cure models were used to fit the OS curves of the extrapolated tislelizumab group and the placebo group, respectively. **(A)** OS curves of the tislelizumab group; **(B)** OS curves of the placebo group; OS, overall survival.

The results of the OS curve fitting extrapolation for the tislelizumab and placebo groups for the Royston/Parmar spline, mixture cure, and non-mixture cure models are shown in **Figure 6**. As shown in **Table 4**, the ICER was lower than the WTP for both models; therefore, tislelizumab in combination with chemotherapy has an economic advantage in the treatment of advanced GC/GEJC. When using the Royston/Parmar spline model, the tislelizumab group had an increase of 0.30 QALYs compared with the placebo group, with an ICER of 33,872.73 USD/QALY; when using the mixture cure model and the non-mixture cure model, the ICERs of the tislelizumab group were 35,560.10 USD/QALY and 33,926.13 USD/QALY.

## 4 Discussions

Immunotherapy is a major breakthrough in the field of cancer treatment that aims to eliminate malignant tumor cells by enhancing the immune defenses of the body, revolutionizing the field of oncology (22). Compared with traditional surgery, radiotherapy, and chemotherapy, tumor immunotherapy has the advantages of good efficacy, tolerability, and few side effects. Immune checkpoint inhibitors are the main methods used in tumor immunotherapy (23). Tislelizumab is an anti-PD-1 monoclonal IgG4 antibody produced independently by China, which can minimize binding to macrophage Fcγ receptor and thus reduce antibody-dependent cell-mediated phagocytosis compared with other PD-1 monoclonal antibodies, as well as counteracting potential resistance to PD-1 therapy (24, 25) It is worth mentioning that tislelizumab in combination with chemotherapy set a new survival record for PD-1 inhibitors in combination with chemotherapy for advanced GC/GEJC, with significant survival benefit.

Innovative antitumor drugs such as PD-1 inhibitors are more expensive than traditional chemotherapeutic agents, which impose a greater economic burden on both patients and the public health system, especially in countries such as China, where healthcare resources are currently relatively limited; thus, cost-effectiveness analyses of new treatment options are necessary. To the best of our knowledge, this study is the first to comprehensively evaluate the economics of tislelizumab in combination with chemotherapy for advanced GC/GEJC from the perspective of China’s health service system, based on the most recent data provided by RATIONALE-305, which showed that tislelizumab in combination with chemotherapy regimens had economic advantages over chemotherapy regimens. Previously Li W et al. (26) also evaluated the economics of tislelizumab combination chemotherapy for advanced GC/GEJC, but at that time RATIONALE-305 did not reveal survival data for the whole population, so Li W et al. only reported the economics for the PD-L1-positive population, and the conclusions were in line with the present study, in addition to the lack of the incidence of adverse events and the proportion of patients receiving post-progression proportion of patients receiving subsequent therapy, and thus the use of data disclosed by other clinical studies as model parameters has some limitations. The data used in this study are more comprehensive and accurate than those used by Li et al., which improves the reliability of the conclusions.

A few PD-1 inhibitors are currently available for advanced GC/GEJC, of which only four are nivolumab, pembrolizumab, sintilimab, and tislelizumab. Prior to the approval of tislelizumab, published literature suggested that nivolumab and pembrolizumab regimens were uneconomical for the treatment of advanced GC/GEJC compared with chemotherapy regimens, whereas the sintilimab regimen yielded the opposite findings (11–13). This may be related to the cost of the drugs, as sintilimab and tislelizumab are included in China’s National Health Insurance Catalog, which has a much lower price (148.51 USD per 100 mg for sintilimab; 180.90 USD per 100 mg for tislelizumab; 1,272.00 USD per 100 mg for nivolumab and 2,463.97 USD per 100 mg for pembrolizumab). Current studies on advanced GC/GEJC have compared PD-1 inhibitors in combination with chemotherapy with chemotherapy alone, and the conclusions drawn are difficult to guide real-world dosing, as in reality, patients and physicians often need to make the best choice of drugs for similar mechanisms in the same disease; therefore, it is important to conduct ‘head-to-head’ comparisons between PD-1 inhibitors. Therefore, a ‘head-to-head’ comparison between PD-1 inhibitors is very important, not only to directly compare their safety and efficacy but also to provide a basis for subsequent economic evaluation.

When evaluating the cost-effectiveness of drugs, scenario analysis is beneficial for better modeling and prediction of real situations and obtaining more reliable and stable economic results. Therefore, this study used two scenarios to evaluate the economics of tislelizumab combination chemotherapy. Scenario analysis 1: The Chinese Guidelines for Pharmacoeconomic Evaluation require that the study timeframe be able to respond to significant differences in cost and effectiveness, but do not make specific requirements for time (14), based on the general survival time of cancer patients, the published literature ultimately assumed the time of 5 and 20 years (27–29), The results showed that as the simulation time period of years was prolonged, the incremental cost and the incremental QALY both increased and ICER decreased, which may be related to the mechanism by which immunosuppressants delay clinical effects. Therefore, the longer the simulation time, the better the clinical effect of tislelizumab combination chemotherapy, and the clinical and economic benefits of this regimen will increase accordingly. Scenario analysis 2: Constructing parametric models Extrapolation is currently one of the most widely used methods in pharmacoeconomic evaluation, in which standard parametric models are usually the most used method for fitting extrapolated survival curves (30). As the follow-up time of clinical trials is not long enough, the long-term survival data of patients are not mature enough, and the immature data will make the survival curves complex, which often manifests as a sudden drop in the survival rate or a plateau period in the tail. For these complex situations, the standard parametric model fit is often inadequate, and is more suitable for survival extrapolation in simple situations. Compared with standard parametric models, Royston/Parmar spline, mixture cure, and non-mixture cure models are relatively flexible (31). The Royston/Parmar spline model is essentially a piecewise polynomial function controlled by some nodes, which fits the extrapolated survival function by adjusting the number and position of nodes. Based on this, the model is better able to adapt to the complexity of the survival function, and in addition to this, the high degree of smoothing between the nodes leads to a smoother final fitted survival curve as well (32, 33). Both mixture cure and non-mixture cure models belong to the cure model, which is a model that divides patients into two parts, the cured and the uncured, and integrates the survival of the overall population through the cure rate, the introduction of the background mortality rate, and the relative survival framework. These types of models are not only capable of grouping patients according to their heterogeneity, but also provide better fitted extrapolation when premised on well-established survival data and reasonable cure rates (31). These three parametric models not only enable a better fit to the survival function, but also further optimize and upgrade the standard parametric model.

Therefore, we used these models to explore the differences in the results of the different parametric models when fitting the extrapolated survival curves. The results of scenario analysis indicated that the Royston/Parmar spline model obtained the closest ICER value (33872.73 USD/QALY) to the standard parametric model. Compared with the other models, the mixture cure model and the non-mixture cure model had higher ICER values of 35560.10 USD/QALY and 33926.13 USD/QALY, respectively. Due to the limited follow-up period of the RATIONALE-305 clinical trial and the relative immaturity of the survival data, it was not possible to determine which model yielded more accurate results for the time being. However, regardless of which model was used, our final results suggest that tislelizumab in combination with chemotherapy is a relatively advantageous regimen for the treatment of advanced GC/GEJC.

This study had several limitations. First, we extrapolated the long-term survival of patients from the K-M curves provided by the RATIONALE-305 trial with some degree of uncertainty; however, this was unavoidable due to the short follow-up period. Second, patient health utility values were not disclosed in the RATIONALE-305 trial; therefore, we obtained PFS and PD state utility values from previously published literature, and the OWSA results indicated that both state utility values had a large impact on ICER. Third, since grade 1 or 2 ADRs are relatively mild, we ignored them in the model. We only considered the most common grade 3 and higher ADRs, which may result in lower ADR costs; however, the OWSA results showed that the incidence of ADRs and the cost of ADRs had a smaller impact on ICER in both groups and did not affect the final outcome.

## 5 Conclusion

From the perspective of the Chinese health service system, tislelizumab combination chemotherapy is more economical than placebo combination chemotherapy for the treatment of advanced GC/GEJC.

## Data Availability

The original contributions presented in the study are included in the article/supplementary material. Further inquiries can be directed to the corresponding authors.
